# Supplementary material for: The interactome of intact mitochondria by cross-linking mass spectrometry provides evidence for coexisting respiratory supercomplexes
Source: Mol Cell Proteomics. 2017 Dec 8;17(2):216–32. doi: 10.1074/mcp.RA117.000470 (PMC5795388; doi:10.1074/mcp.RA117.000470)
Supplement: Supplemental Data [file supp_RA117.000470_133922_0_supp_23978_fzffwf.zip › spectra_annotation/mito_DR_spectra_annotation/142-1-2-1-4-1.pdf]

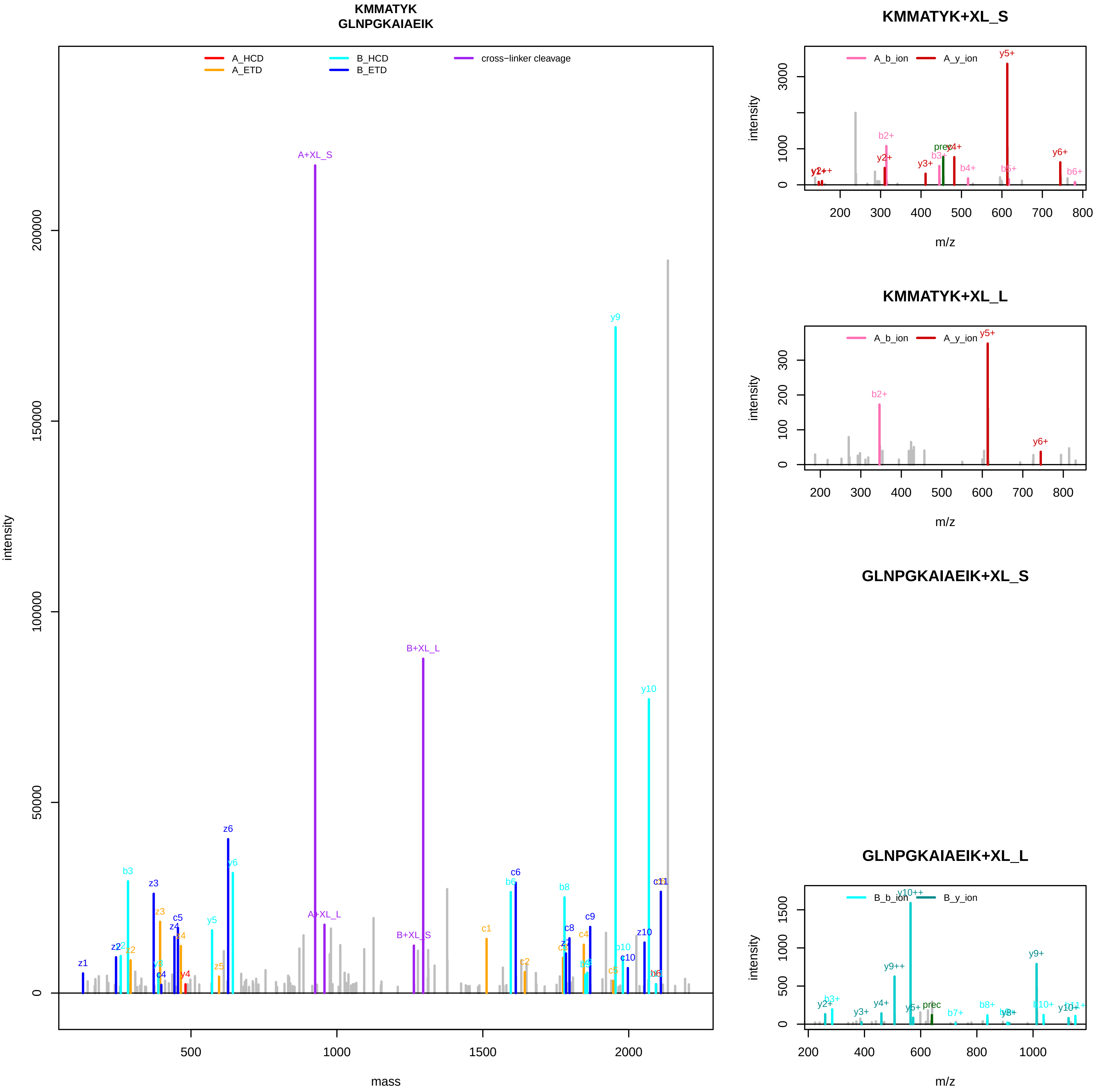
KMMATYK
GLNPGKAIAEIK
A_HCD
A_ETD
B_HCD
B_ETD
cross−linker cleavage
A+XL_S
B+XL_L
A+XL_L
B+XL_S
y9
y10
z1
z2
z3
z4
z5
z6
z10
c1
c2
c3
c4
c5
c6
c8
c9
c10
c11
b3
b6
b8
y3
y4
y5
y6
intensity
mass
0
50000
100000
150000
200000
500
1000
1500
2000
KMMATYK+XL_S
A_b_ion
A_y_ion
y5+
y6+
y4+
y3+
y2+
b2+
b3+
b4+
b5+
b6+
prec
intensity
m/z
0
1000
3000
200
300
400
500
600
700
800
KMMATYK+XL_L
A_b_ion
A_y_ion
y5+
y6+
b2+
intensity
m/z
0
100
200
300
200
300
400
500
600
700
800
GLNPGKAIAEIK+XL_S
GLNPGKAIAEIK+XL_L
B_b_ion
B_y_ion
y10++
y9++
y9+
y2+
b3+
y3+
y4+
y5+
prec
b7+
b8+
b10+
y10+
intensity
m/z
0
500
1000
1500
200
400
600
800
1000
